# Supplementary material for: Resolving the full spectrum of human genome variation using Linked-Reads
Source: Genome Res. 2019 Apr;29(4):635–45. doi: 10.1101/gr.234443.118 (PMC6442396; doi:10.1101/gr.234443.118)
Supplement: Supplemental Material [file supp_gr.234443.118_Supplemental_longranger-2.2.zip › longranger-2.2.tar.bz2!/lr2.2-public/lib/stan/cmdstan/stan/doc/api/html/structstan_1_1gm_1_1expression.html]

Stan: stan::gm::expression Struct Reference


|  |  |
| --- | --- |
|  | Stan  2.5.0  probability, sampling & optimization |


- Main Page
- Related Pages
- Namespaces
- Classes
- Files

- Class List
- Class Index
- Class Hierarchy
- Class Members

All Classes Namespaces Files Functions Variables Typedefs Enumerations Enumerator Friends Macros Pages

- stan
- gm
- expression

Public Types |
Public Member Functions |
Public Attributes |
List of all members

stan::gm::expression Struct Reference

`#include <ast.hpp>`

|  |  |
| --- | --- |
| Public Types | |
| typedef boost::variant  < boost::recursive\_wrapper  < nil >  , boost::recursive\_wrapper  < int\_literal >  , boost::recursive\_wrapper  < double\_literal >  , boost::recursive\_wrapper  < array\_literal >  , boost::recursive\_wrapper  < variable >  , boost::recursive\_wrapper  < integrate\_ode >  , boost::recursive\_wrapper  < fun >  , boost::recursive\_wrapper  < index\_op >  , boost::recursive\_wrapper  < binary\_op >  , boost::recursive\_wrapper  < unary\_op > > | expression\_t |
|  | |

|  |  |
| --- | --- |
| Public Member Functions | |
|  | expression () |
|  | |
|  | expression (const expression &e) |
|  | |
|  | expression (const nil &expr) |
|  | |
|  | expression (const int\_literal &expr) |
|  | |
|  | expression (const double\_literal &expr) |
|  | |
|  | expression (const array\_literal &expr) |
|  | |
|  | expression (const variable &expr) |
|  | |
|  | expression (const fun &expr) |
|  | |
|  | expression (const integrate\_ode &expr) |
|  | |
|  | expression (const index\_op &expr) |
|  | |
|  | expression (const binary\_op &expr) |
|  | |
|  | expression (const unary\_op &expr) |
|  | |
|  | expression (const expression\_t &expr\_) |
|  | |
| expr\_type | expression\_type () const |
|  | |
| expression & | operator+= (const expression &rhs) |
|  | |
| expression & | operator-= (const expression &rhs) |
|  | |
| expression & | operator\*= (const expression &rhs) |
|  | |
| expression & | operator/= (const expression &rhs) |
|  | |

|  |  |
| --- | --- |
| Public Attributes | |
| expression\_t | expr\_ |
|  | |

## Detailed Description

Definition at line 216 of file ast.hpp.

## Member Typedef Documentation

typedef boost::variant<boost::recursive\_wrapper<nil>, boost::recursive\_wrapper<int\_literal>, boost::recursive\_wrapper<double\_literal>, boost::recursive\_wrapper<array\_literal>, boost::recursive\_wrapper<variable>, boost::recursive\_wrapper<integrate\_ode>, boost::recursive\_wrapper<fun>, boost::recursive\_wrapper<index\_op>, boost::recursive\_wrapper<binary\_op>, boost::recursive\_wrapper<unary\_op> > stan::gm::expression::expression\_t

Definition at line 227 of file ast.hpp.

## Constructor & Destructor Documentation

|  |  |  |  |  |
| --- | --- | --- | --- | --- |
| stan::gm::expression::expression | ( |  | ) |  |

Definition at line 564 of file ast\_def.cpp.

|  |  |  |  |  |  |
| --- | --- | --- | --- | --- | --- |
| stan::gm::expression::expression | ( | const expression & | *e* | ) |  |

Definition at line 567 of file ast\_def.cpp.

|  |  |  |  |  |  |
| --- | --- | --- | --- | --- | --- |
| stan::gm::expression::expression | ( | const nil & | *expr* | ) |  |

Definition at line 578 of file ast\_def.cpp.

|  |  |  |  |  |  |
| --- | --- | --- | --- | --- | --- |
| stan::gm::expression::expression | ( | const int\_literal & | *expr* | ) |  |

Definition at line 579 of file ast\_def.cpp.

|  |  |  |  |  |  |
| --- | --- | --- | --- | --- | --- |
| stan::gm::expression::expression | ( | const double\_literal & | *expr* | ) |  |

Definition at line 580 of file ast\_def.cpp.

|  |  |  |  |  |  |
| --- | --- | --- | --- | --- | --- |
| stan::gm::expression::expression | ( | const array\_literal & | *expr* | ) |  |

Definition at line 581 of file ast\_def.cpp.

|  |  |  |  |  |  |
| --- | --- | --- | --- | --- | --- |
| stan::gm::expression::expression | ( | const variable & | *expr* | ) |  |

Definition at line 582 of file ast\_def.cpp.

|  |  |  |  |  |  |
| --- | --- | --- | --- | --- | --- |
| stan::gm::expression::expression | ( | const fun & | *expr* | ) |  |

Definition at line 584 of file ast\_def.cpp.

|  |  |  |  |  |  |
| --- | --- | --- | --- | --- | --- |
| stan::gm::expression::expression | ( | const integrate\_ode & | *expr* | ) |  |

Definition at line 583 of file ast\_def.cpp.

|  |  |  |  |  |  |
| --- | --- | --- | --- | --- | --- |
| stan::gm::expression::expression | ( | const index\_op & | *expr* | ) |  |

Definition at line 585 of file ast\_def.cpp.

|  |  |  |  |  |  |
| --- | --- | --- | --- | --- | --- |
| stan::gm::expression::expression | ( | const binary\_op & | *expr* | ) |  |

Definition at line 586 of file ast\_def.cpp.

|  |  |  |  |  |  |
| --- | --- | --- | --- | --- | --- |
| stan::gm::expression::expression | ( | const unary\_op & | *expr* | ) |  |

Definition at line 587 of file ast\_def.cpp.

|  |  |  |  |  |  |
| --- | --- | --- | --- | --- | --- |
| stan::gm::expression::expression | ( | const expression\_t & | *expr\_* | ) |  |

Definition at line 577 of file ast\_def.cpp.

## Member Function Documentation

|  |  |  |  |  |
| --- | --- | --- | --- | --- |
| expr\_type stan::gm::expression::expression\_type | ( |  | ) | const |

Definition at line 570 of file ast\_def.cpp.

|  |  |  |  |  |  |
| --- | --- | --- | --- | --- | --- |
| expression & stan::gm::expression::operator\*= | ( | const expression & | *rhs* | ) |  |

Definition at line 1396 of file ast\_def.cpp.

|  |  |  |  |  |  |
| --- | --- | --- | --- | --- | --- |
| expression & stan::gm::expression::operator+= | ( | const expression & | *rhs* | ) |  |

Definition at line 1386 of file ast\_def.cpp.

|  |  |  |  |  |  |
| --- | --- | --- | --- | --- | --- |
| expression & stan::gm::expression::operator-= | ( | const expression & | *rhs* | ) |  |

Definition at line 1391 of file ast\_def.cpp.

|  |  |  |  |  |  |
| --- | --- | --- | --- | --- | --- |
| expression & stan::gm::expression::operator/= | ( | const expression & | *rhs* | ) |  |

Definition at line 1401 of file ast\_def.cpp.

## Member Data Documentation

expression\_t stan::gm::expression::expr\_

Definition at line 252 of file ast.hpp.

---

The documentation for this struct was generated from the following files:

- src/stan/gm/ast.hpp
- src/stan/gm/ast\_def.cpp


---

[ Stan Home Page ]

*© 2011–2014,
Stan Development Team.*
